# Impact of meningoencephalitis and sepsis on delirium and subsequent neurological impairment in pediatric patients: a prospective proof-of-concept biomarker and EEG study

**DOI:** 10.1038/s41598-025-31058-2

**Published:** 2025-12-08

**Authors:** Felix Klawitter, Johanna Hackenberg, Lena Danckert, Astrid Bertsche, Axel Petzold, Heidi A.B. Smith, Eugene Wesley Ely, Christian Spang, Rika Bajorat, Dagmar-Christiane Fischer, Gerd Klinkmann, Anne Körmann-Günther, Robert Fleischmann, Amanda Heslegrave, Henrik Zetterberg, Hessel Peters-Sengers, Johannes Ehler

**Affiliations:** 1 Department of Anesthesiology, Intensive Care Medicine and Pain Therapy, Interdisciplinary Paediatric Intensive Care Medicine, University Medical Centre Rostock, Rostock, Germany; 2 Department of Paediatrics, Interdisciplinary Paediatric Intensive Care Medicine, University Medical Centre Rostock, Rostock, Germany; 3 Department of Neuropaediatrics, University Medical Centre Greifswald, Greifswald, Germany; 4 The National Hospital for Neurology and Neurosurgery, University College London & Moorfields Eye Hospital, London, UK; 5 Department of Paediatrics, Children’s Hospital, Vanderbilt University Medical Centre, Monroe Carell Jr, Nashville, USA; 6 Department of Anaesthesiology, Vanderbilt University Medical Centre, Nashville, USA; 7 Critical Illness, Brain Dysfunction, Survivorship (CIBS) Centre, Department of Medicine, Vanderbilt University Medical Centre, Nashville, USA; 8 Tennessee Valley Veteran’s Affairs Geriatric Research Education Clinical Centre (GRECC), Nashville, USA; 9 Department of Extracorporeal Therapy Systems, Fraunhofer Institute for Cell Therapy and Immunology, Rostock, Germany; 10 International Renal Research Institute of Vicenza, Vicenza, Veneto, Italy; 11 Department of Neurology, University Medicine Greifswald, Greifswald, Germany; 12 UK Dementia Research Institute at University College London, Fluid Biomarker Laboratory, London, WC1E 6BT UK; 13 Department of Neurodegenerative Disease, UCL Queen Square Institute of Neurology, University College London, London, WC1N 3BG UK; 14 Department of Psychiatry and Neurochemistry, Institute of Neuroscience and Physiology, the Sahlgrenska Academy at the University of Gothenburg, Mölndal, Sweden; 15 Clinical Neurochemistry Laboratory, Sahlgrenska University Hospital, Mölndal, Sweden; 16 Hong Kong Center for Neurodegenerative Diseases, InnoHK, Hong Kong China; 17 Wisconsin Alzheimer’s Disease Research Center, School of Medicine and Public Health, University of Wisconsin, University of Wisconsin- Madison, Madison, WI 53792 USA; 18 Center for Infection and Molecular Medicine, Location AMC, Amsterdam University Medical Center, University of Amsterdam, Amsterdam, Netherlands; 19 Department of Epidemiology and Data Science, Amsterdam UMC location Vrije Universiteit Amsterdam, Amsterdam, Netherlands; 20 Department of Anaesthesiology and Intensive Care Medicine, University Hospital Jena, Am Klinikum 1, 07749 Jena, Germany

**Keywords:** Delirium, Children, Intensive care unit, Encephalopathy, Brain injury, Body fluid biomarkers, Biomarkers, Diseases, Risk factors

## Abstract

To assess the incidence and severity of delirium and subsequent neurological impairment in pediatric intensive care unit (PICU) patients with meningoencephalitis (ME) and sepsis. Prospective study enrolling PICU patients with sepsis or meningoencephalitis (ME) and healthy controls. Daily delirium assessment, repeated electroencephalography, blood sampling at days 1, 3, 5 and lumbar puncture, if clinically indicated, for cerebrospinal fluid (CSF) sampling were performed. Biomarkers of inflammation (CRP, PCT, IL-6), endothelial activation (NT-proCNP), neurodegeneration (NSE, tau), neuroaxonal damage (NfL, NfH, UCHL-1) and glial injury (GFAP, S100B) were measured. Neurological status was assessed using the Functional Status Scale (FSS), the pediatric overall performance category (POPC) and the pediatric cerebral performance scale (PCPC) before admission and after three months. Twenty-four PICU patients (ME *n* = 9, sepsis *n* = 15) and eleven controls were enrolled. The delirium incidence was not statistically higher in patients with sepsis (60%, *n* = 9/15) compared to ME (33%, *n* = 3/9, *p* = 0.105). Children with delirium (*n* = 12) had a higher FSS (10.0 [9.8, 12.0] vs. 7.5 [7.0, 9.0], *p* = 0.009) at day 1 than children without delirium (*n* = 12), without significant differences in blood and CSF biomarker levels. Patients with ME presented higher levels of blood NfL compared to sepsis patients at day 3 (16.8 [12.9, 77.5] vs. 10.7 [8.6, 12.2] pg/ml, *p* = 0.011) and day 5 (16.2 [6.8, 170.4] vs. 12.0 [7.9, 14.3] pg/ml, *p* = 0.005). Routine EEG results did not differ between the groups, but quantitative EEG parameters representing encephalopathy correlated with biomarkers of brain injury. FSS, POPC and PCPC after three months were identical between in all patients. Independent from the origin of inflammatory stimulation by sepsis or ME delirium is associated with transient neurological dysfunction in PICU patients, without neurological impairment after three months, as confirmed by biomarker levels.

**Supplementary Information:**

The online version contains supplementary material available at 10.1038/s41598-025-31058-2.

## Background

Delirium is one of the most frequent neuropsychiatric complications in critically ill patients and can be triggered by multiple causes, including infection, trauma and critical illness itself. ^1^ Typically, delirium is characterized by an acute and transient change in behavior, awareness and other cognitive abilities, but neurocognitive impairments may persist for months, increasing morbidity and mortality^4,5^. In daily clinical routine delirium is challenging due to its complex presentation. Particularly in special populations like critically ill pediatric patients, delirium is often underdiagnosed^6^. Therefore, validated biomarkers of brain injury might help to close the gap between patient-related diagnostic short-comings and the detection of neurocognitive impairment in a highly vulnerable patient cohort. ^2,7^ It has been estimated, that delirium affects between 25 and 60% of patients in the pediatric intensive care unit (PICU)^10,11^. Similar to adults symptoms can persist with varying duration, worsening patient outcome^11^. Beyond the acute phase of illness, delirium in pediatric patients has also been associated with impairments in post-discharge cognition and quality of life, indicating persistent central nervous system (CNS) injury^12^. However, data regarding the extent of severe neurological impairment and consecutive deleterious long-term outcomes in pediatric patients are limited^13,14^.

In this proof-of-concept study, we hypothesized that critically ill pediatric patients with delirium exhibit clinical and laboratory features of structural brain injury resulting in subsequent neurological impairment compared to critically ill children without delirium. Furthermore, we hypothesized that the source of infection, represented by either systemic infection (sepsis) with secondary involvement of the brain and primary infection (ME) is relevant for the development of delirium as well as for structural brain injury. Finally, we hypothesized that routine electroencephalography (EEG) improves the detection of encephalopathy in pediatric patients with delirium and EEG results correlate with brain injury biomarker levels.

## Materials and methods

### Study design, setting, ethical approval and trial registration

We conducted a prospective observational single center cohort study in pediatric patients treated at a university hospital. The study was approved by the local ethics committee of Rostock University (identifier A 2020 − 0160) and was prospectively registered (clinicaltrials.gov: NCT04467762, 08-JUL 2020). The STROBE guidelines apply. Furthermore, all diagnostic and therapeutic procedures were performed in accordance with pediatric clinical guidelines and the Declaration of Helsinki.

### Inclusion and exclusion criteria

Pediatric patients between one day and 17 years of age with an acute severe infection (symptom duration below 24 h) admitted to the PICU at the University Medical Center Rostock were eligible. Pediatric sepsis definition based on the International pediatric sepsis consensus conference 2005 criteria used at the time of patient recruitment^15^. These criteria comprised at least two SIRS criteria plus suspicion or evidence of infection^15^. Children with clinical signs of ME, e.g. fever, altered level of consciousness, abnormal behaviour, headache, meningeal irritation, new focal neurologic deficits or seizures and with pleocytosis in the CSF (with or without microbiological detection of pathogens), were defined to have ME^16^.

Written informed consent was given by legal representatives before study inclusion. Exclusion criteria comprised preexisting CNS diseases (e.g. stroke, epilepsy or tumor), immunosuppression and participation in another study. Additionally, pediatric patients with hospital admission for minor elective surgery (e.g. material removal after osteosynthesis or surgery for hernia inguinalis) but without acute infection or above-mentioned exclusion criteria were recruited as healthy controls.

### Study visits, sampling and data collection

**Fig. 1:**
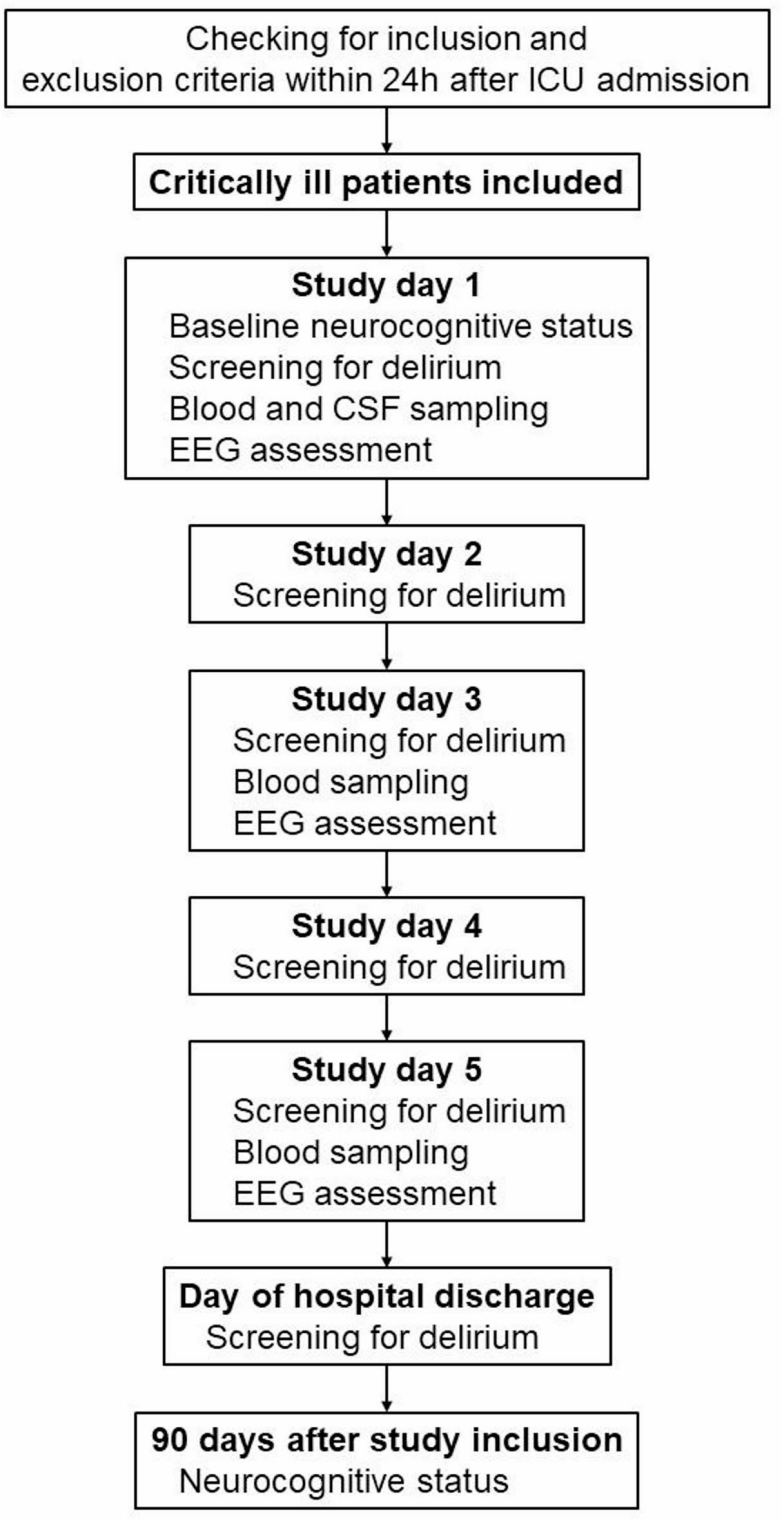
Study flow chart. CSF - Cerebrospinal fluid; EEG - Electroencephalography.

Study visits with neurological examinations and delirium assessment were performed daily within the first five days after study inclusion and at the time of hospital discharge by experienced study personnel (Fig. 1). Blood sampling and EEG recording were performed at study days 1 (day of enrolment), 3 and 5. Lumbar puncture and cerebrospinal fluid (CSF) sampling was performed once at study day 1, if clinically indicated. Three months after enrolment, patients received a detailed neurological follow-up examination or, if indisposed, standardized telephone interviews. Demographic and clinical parameters were collected using the in-house patient data management system. The Functional status scale (FSS), the Glasgow Coma Scale (GCS), the Richmond Agitation and Sedation Scale (RASS) and the pediatric Sequential Organ Failure Assessment (pSOFA) Score were documented at each study visit.

Healthy controls received the same clinical assessments like patients at day 1 during their preanesthetic visit (e.g. demographic data, neurofunctional tests, delirium screening tests, but no EEG). Blood sampling was performed in the operating room directly after placing the intravenous access before the induction of anesthesia.

### Assessment of delirium and neurological outcome

Delirium was assessed using the pediatric Confusion Assessment Method (pCAM) for the ICU (for patients six years or older), the preschool (ps)CAM-ICU (between six months and five years) and the Sophia Observation Withdrawal Symptoms-scale for pediatric delirium (SOS-PD Scale) at all study visits once a day. Additionally, all patients including patients below six months of age were clinically assessed for signs of delirium by a neuropediatrician (AB). Furthermore, nursing reports were screened for signs of delirium outside the assessment procedures to reduce the risk of missed delirium events.

Three months after enrolment, patients underwent a comprehensive neurological examination including neurological status, POPC, PCPC, FSS and established developmental testing, which were compared to the pre-hospital developmental status^17–20^. For children aged 0 to 6 months, the Münchener Funktionelle Entwicklungsdiagnostik (MFED) was used, while children aged 6 months to 4 years were assessed with the Entwicklungstest für Kinder von 6 Monaten bis 6 Jahren (ET 6-6-R). For children older than 4 years, the Kaufman Assessment Battery for Children (KABC-II) was applied. For patients that were not available for in-house follow-up examination (due to restrictions in line with the COVID pandemia) a standardized telephone interview was performed. This consisted of detailed information provided by patients’ caregivers on the clinical and neurological development including potential residual symptoms as well as an assessment using the POPC, PCPC and FSS.

### Electroencephalography

EEG was performed using the 10–20 system with bipolar montages and 20 min recording time to assess the severity of encephalopathy in the context of delirium (Neurofax EEG-1200, Nihon Kohden, Japan). If applicable, sedation was interrupted prior to recording. Normal EEGs were represented by physiological age-dependent background activity and the absence of pathological discharges. Abnormal EEGs were defined by either background activity deviating from the age norm and/or pathological discharges.

Further quantitative EEG (qEEG) processing was conducted using Brainwave (Version 0.9.165.57, freely available at http://home.kpn.nl/stam7883/brainwave.html). Analyses were stratified by age groups (1–11 months, 1–8 years, and 9–18 years) to account for developmental changes in qEEG features during brain maturation. Data was referenced to an average reference montage and power spectral analysis was performed using a Fast Fourier Transform. Power spectra and connectivity measures were calculated for delta (0.5–4 Hz), theta (4–8 Hz), alpha (8–13 Hz) and lower beta (13–20 Hz) bands. Band-pass filtering to lower beta frequencies was done to mitigate the impact of muscle activity^21^. The phase lag index (PLI) was used to calculate the functional connectivity strength^22^. The PLI is derived from the instantaneous phase differences obtained through the Hilbert transform, capturing the asymmetry between phase leading and lagging of two signals. PLI values range from 0 to 1, where 1 indicates perfect phase locking, and 0 signifies no phase synchronization or equal amounts of leading and lagging over the epoch. For each epoch, the PLI values across all channel pairs were averaged to compute a mean PLI for each frequency band per subject. Amplitude envelope correlation (AEC) was used to assess amplitude coupling between signals. This metric represents the Pearson correlation between the amplitude envelopes of the signals, which are derived via the Hilbert transform of the time series. In this study, we utilized the corrected version (AECc) to account for spatial leakage or volume conduction^23^. The correction for volume conduction is achieved by pairwise orthogonalization between signals X and Y (in both directions X to Y and Y to X) before calculation of the Pearson correlation coefficient, as described before^24^. The positive part of the correlation coefficient was used to estimate connectivity strength, which ranges between 0 and 1. ^24^

### Body-fluid biomarkers

Eleven blood-based and five cerebrospinal fluid biomarkers were measured. Biomarkers of inflammation (C-reactive protein [CRP], Procalcitonin [PCT], Interleukin-6 [IL-6 ]), endothelial activation (N-Terminal pro C-type natriuretic neptide [NT-proCNP]), global neurodegeneration (Neuro-specific enolase [NSE], tau), neuroaxonal damage (Neurofilament light chains [NfL], Neurofilament heavy chains [NfH], Ubiquitin-Carboxyl-Terminal-Hydrolase [UCHL)-1]) and glial injury (glial fibrillary acidic protein [GFAP], S100 calcium-binding protein B *(*S100B)] were assessed.

Blood (plasma and serum) and CSF samples were centrifuged at 2,000 g (4 °C) for 15 min, aliquoted and stored at -80 °C until analysis. NfH (R-PLEX Human Neurofilament H Assay) was measured with electrochemiluminescence-based immunoassays (ECLIA) according to manufacturer’s recommendations using the MESO Quickplex SQ120 (Meso Scale Discovery, Rockville, MD, USA). GFAP, NfL, tau protein and UCHL-1 concentrations were measured using Single molecule array (Simoa, Neurology 4-Plex) assays on an HD-1 Analyzer (Quanterix, Billerica, MA, USA), as described before. ^25^ S100B (Cloud-Clone Corp., Houston, USA) and NT-proCNP (Biomedica Medizinprodukte GmbH, Vienna, Austria) were quantified using sandwich enzyme-linked immunosorbent assays (ELISA). NSE concentration was measured using ECLIA in the certified laboratory of the Rostock University Medical Center.

All biomarker samples were measured in duplicates. For samples with a coefficient of variation (CV) > 20%, we repeated the measurement. No results were excluded.

### Statistical analysis

Data were analyzed using R studio built (R version 4.4.1, R Core Team 2013, Vienna, Austria). Categorical variables were compared with the Chi²-Test or the Fisher’s exact test, if the expected probability was < 5%. Continuous variables were compared by the Wilcoxon signed-rank test. Biomarker levels were logarithmically transformed and analyzed using linear mixed models (LMM). The LMM was fitted taking the group (delirium vs. no delirium, meningoencephalitis vs. sepsis, abnormal EEG vs. normal EEG), the time point (study visits 1, 3 and 5) and their interaction term (group x time point) as fixed effects and patient-specific intercepts as random. The interaction term was used to reveal differences in biomarker trajectories over time between the corresponding groups. The patient-specific intercept accounted for repeated measurements at the study visits 1, 3 and 5 within each patient. As bespoke biomarker levels can be confounded by age, we additionally performed a separate analysis including age as covariate into the LMM. Exploratory analyses were conducted to assess the correlation between qEEG parameters indicative of encephalopathic changes and the maximum or mean values of fluid biomarkers that showed alterations during treatment. Pearson’s correlation coefficient was used, with adjustments for multiple comparisons using the Benjamini-Hochberg false discovery rate procedure. A *p* < 0.05 (two sided) was considered statistically significant.

## Results

Between July 2020 and December 2021, a total of 1044 patients were admitted to the PICU and were screened for participation in the study. Based on the in- and exclusion criteria 998 patients were not eligible for study participation. From 46 eligible patients, written informed consent from the legal representatives could not be achieved in 22 cases, resulting in 24 PICU patients and 11 healthy controls who were enrolled (Fig. 1; Table 1). Focused sub-group analyses have been performed between patients and healthy controls, PICU patients with delirium and without delirium, PICU patients with ME and sepsis as well as PICU patients with abnormal and normal EEG. All corresponding groups were comparable for age and sex. Patients with sepsis were significantly younger than patients with ME (17.0 [5.5, 55.0] months vs. 99.0 [64.0, 151.0] months, *p* = 0.032). Furthermore, septic patients (2.0 [1.0, 2.5] vs. 0.0 [0.0, 0.0], *p* = 0.001) and patients with delirium (1.5 [1.0, 2.0] vs. 0.0 [0.0, 1.2], *p* = 0.047) had higher pSOFA scores at day 1.

**Table 1:** Demographics and clinical characteristics of pediatric patients.

	Patients	Controls	*p* value	Meningo-encephalitis	Sepsis	*p* value	Delirium	No delirium	*p* value	Abnormal EEG	Normal EEG	*p* value
Number (n)	24	11		9	15		12	12		8	16	
Age (months)	34.0 [8.2,99.2]	59.0 [35.5,79.0]	0.845	99.0 [64.0,151.0]	17.0 [5.5,55.0]	0.032	21.0 [8.0,45.2]	95.5 [17.2,151.2]	0.133	62.5 [8.0,163.2]	34.0 [9.8,77.0]	0.581
Female (n,%)	12 (50.0%)	2 (18.2%)	0.137	3 (33.3%)	9 (60.0%)	0.976	7 (58.3%)	5 (41.7%)	0.684	5 (62.5%)	7 (43.8%)	0.667
Male	12 (50.0%)	9 (81.8%)		6 (66.7%)	6 (40.0%)		5 (41.7%)	7 (58.3%)		3 (37.5%)	9 (56.3%)	
BMI (kg/m²)	16.2 [14.9,17.7]	16.2 [14.9,17.0]	0.790	16.1 [15.4,17.6]	16.4 [14.8,17.6]	0.976	16.5 [14.8,17.5]	16.2 [15.3,18.7]	0.885	19.6 [16.7,22.4]	15.8 [14.8,16.6]	0.037
pSOFA day 1	1.0 [0.0,2.0]	0.0 [0.0,0.0]	0.001	0.0 [0.0,0.0]	2.0 [1.0,2.5]	0.01	1.5 [1.0,2.0]	0.0 [0.0,1.2]	0.047	2.0 [0.8,2.2]	1.0 [0.0,1.2]	0.161
pSOFA day 3	0.0 [0.0,0.0]	NA	NA	0.0 [0.0,0.0]	0.0 [0.0,0.0]	0.50	0.0 [0.0,0.0]	0.0 [0.0,0.0]	1.000	0.0 [0.0,1.5]	0.0 [0.0,0.0]	0.053
pSOFA day 5	0.0 [0.0,0.0]	NA	NA	0.0 [0.0,0.0]	0.0 [0.0,0.0]	1.000	0.0 [0.0,0.0]	0.0 [0.0,0.0]	0.698	0.0 [0.0,0.2]	0.0 [0.0,0.0]	0.276
Length of hospital stay (days)	7.0 [6.0,9.5]	NA	NA	6.0 [5.0,8.0]	8.0 [7.0,9.0]	0.131	8.0 [7.0,11.8]	6.5 [4.8,8.0]	0.035	8.0 [7.0,9.0]	7.0 [5.0,8.5]	0.137
Length of ICU stay (days)	4.0 [4.0,7.0]	NA	NA	0.0 [0.0,2.0]	5.0 [3.5,7.5]	0.016	5.5 [2.8,8.8]	1.0 [0.0,4.2]	0.087	5.0 [1.5,9.0]	4.0 [0.0,5.5]	0.452
Pre-admission FSS	6.0 [6.0,6.0]	6.0 [6.0,6.0]	NA	6.0 [6.0,6.0]	6.0 [6.0,6.0]	NA	6.0 [6.0,6.0]	6.0 [6.0,6.0]	NA	6.0 [6.0,6.0]	6.0 [6.0,6.0]	NA
Pre-admission POPC	1.0 [1.0,1.0]	1.0 [1.0,1.0]	NA	1.0 [1.0,1.0]	1.0 [1.0,1.0]	NA	1.0 [1.0,1.0]	1.0 [1.0,1.0]	NA	1.0 [1.0,1.0]	1.0 [1.0,1.0]	NA
Pre-admission PCPC	1.0 [1.0,1.0]	1.0 [1.0,1.0]	NA	1.0 [1.0,1.0]	1.0 [1.0,1.0]	NA	1.0 [1.0,1.0]	1.0 [1.0,1.0]	NA	1.0 [1.0,1.0]	1.0 [1.0,1.0]	NA
Analgosedation (days)	0.0 [0.0,0.4]	NA	NA	0.0 [0.0,0.0]	0.0 [0.0,0.0]	0.414	0.0 [0.0,0.0]	0.0 [0.0,0.0]	0.692	0.0 [0.0,0.1]	0.0 [0.0,0.0]	0.061
Delirium (n,%)	12 (50%)	0 (0%)	0.006	3 (33.3%)	9 (60%)	0.105	NA	NA	NA	4 (50%)	8 (50%)	1.0

BMI = Body mass index; FSS = Functional status scale; ICU = Intensive care unit; n = number; NA = not accessible; PCPC = Pediatric cerebral performance category scale; POPC = Pediatric overall performance category pSOFA = Pediatric sequential organ failure assessment score. Values are given as medians [interquartile range].

Continuous analgosedation was present in one patient with sepsis receiving invasive mechanical ventilation, with successful weaning and extubation nine hours after admission at day 1. Periprocedural short-term analgosedation was used for lumbar puncture at day 1 in two children with ME and four children with sepsis at day 1.

After three months, 58.3% (*n* = 14/24) of PICU patients were available for clinical in-house follow-up examination, 41.7% (*n* = 10/24) were evaluated via standardized telephone interviews. All patients survived until three months after study inclusion without neurological impairment (Table 2).

**Table 2:** Neurological assessment of pediatric patients.

	Patients	Controls	*p* value	Meningoencephalitis	Sepsis	*p* value	Delirium	No delirium	*p* value	Abnormal EEG	Normal EEG	*p* value
Number (n)	24	11		9	15		12	12		8	16	
FSS day 1	9.0 [7.0,10.2]	6.0 [6.0,6.5]	< 0.001	7.0 [7.0,9.0]	10.0 [8.5,11.5]	0.019	10.0 [9.8,12.0]	7.5 [7.0,9.0]	0.009	9.0 [7.8,12.0]	9.5 [7.0,10.0]	0.577
FSS day 3	7.0 [6.8,7.2]	NA	NA	7.0 [6.0,7.0]	7.0 [7.0,9.0]	0.198	7.0 [7.0,9.0]	7.0 [6.0,7.0]	0.120	7.0 [7.0,9.5]	7.0 [6.0,7.0]	0.176
FSS day 5	6.0 [6.0,7.0]	NA	NA	7.0 [6.0,7.0]	6.0 [6.0,7.0]	0.697	6.0 [6.0,7.2]	6.5 [6.0,7.0]	1.0	7.0 [6.0,7.2]	6.0 [6.0,7.0]	0.242
GCS day 1	15.0 [13.8,15.0]	15.0 [15.0,15.0]	0.009	15.0 [15.0,15.0]	14.0 [13.0,15.0]	0.042	13.5 [13.0,14.0]	15.0 [15.0,15.0]	< 0.001	14.5 [12.8,15.0]	15.0 [14.0,15.0]	0.459
GCS day 3	15.0 [15.0,15.0]	NA	NA	15.0 [15.0,15.0]	15.0 [15.0,15.0]	0.804	15.0 [15.0,15.0]	15.0 [15.0,15.0]	0.166	15.0 [15.0,15.0]	15.0 [15.0,15.0]	0.610
GCS day 5	15.0 [15.0,15.0]	NA	NA	15.0 [15.0,15.0]	15.0 [15.0,15.0]	0.621	15.0 [15.0,15.0]	15.0 [15.0,15.0]	0.209	15.0 [15.0,15.0]	15.0 [15.0,15.0]	0.732
RASS day 1	0.0 [-1.0,0.0]	0.0 [0.0,0.0]	0.266	0.0 [0.0,0.0]	0.0 [-1.0,0.5]	0.799	-1.0 [-1.0,0.2]	0.0 [0.0,0.0]	0.096	0.0 [-1.2,0.2]	0.0 [-1.0,0.0]	0.870
RASS day 3	0.0 [0.0,0.0]	NA	NA	0.0 [0.0,0.0]	0.0 [0.0,0.0]	0.710	0.0 [-1.0,0.0]	0.0 [0.0,0.0]	0.264	0.0 [0.0,0.0]	0.0 [0.0,0.0]	0.566
RASS day 5	0.0 [0.0,0.0]	NA	NA	0.0 [0.0,0.0]	0.0 [0.0,0.0]	0.442	0.0 [0.0,0.0]	0.0 [0.0,0.0]	0.619	0.0 [0.0,0.0]	0.0 [0.0,0.0]	0.492
POPC day 90	1.0 [1.0,1.0]	NA	NA	1.0 [1.0,1.0]	1.0 [1.0,1.0]	0.756	1.0 [1.0,1.0]	1.0 [1.0,1.0]	1.0	1.0 [1.0,1.0]	1.0 [1.0,1.0]	0.655
PCPC day 90	1.0 [1.0,1.0]	NA	NA	1.0 [1.0,1.0]	1.0 [1.0,1.0]	NA	1.0 [1.0,1.0]	1.0 [1.0,1.0]	NA	1.0 [1.0,1.0]	1.0 [1.0,1.0]	NA
FSS day 90	6.0 [6.0,6.0]	NA	NA	6.0 [6.0,6.0]	6.0 [6.0,6.0]	NA	6.0 [6.0,6.0]	6.0 [6.0,6.0]	NA	6.0 [6.0,6.0]	6.0 [6.0,6.0]	NA
RASS day 90	0.0 [0.0,0.0]	NA	NA	0.0 [0.0,0.0]	0.0 [0.0,0.0]	NA	0.0 [0.0,0.0]	0.0 [0.0,0.0]	NA	0.0 [0.0,0.0]	0.0 [0.0,0.0]	NA
GCS day 90	15.0 [15.0,15.0]	NA	NA	15.0 [15.0,15.0]	15.0 [15.0,15.0]	1.0	15.0 [15.0,15.0]	15.0 [15.0,15.0]	1.0	15.0 [15.0, 15.0]	15.0 [15.0,15.0]	NA

FSS = Functional status scale; GCS = Glasgow coma score; NA = Not accessible; PCPC = Pediatric cerebral performance scale; POPC = Pediatric overall performance scale; RASS = Richmond agitation and sedation scale. Values are given as medians [interquartile range].

### Patients versus controls

Biomarkers of systemic inflammation [white blood count (WBC), CRP and PCT, IL-6] were significantly higher in patients than controls (eTable 1). NSE was significantly lower in patients at day 1 (781.2 [529.2, 991.7] pg/ml) compared to controls (1760.4 [1216.5, 2550.7] pg/ml, *p* < 0.001). UCHL-1 was significantly higher in patients at day 1 (22.2 [14.9, 37.8] ng/ml) than in controls (14.2 [6.0, 20.7] ng/ml, *p* = 0.032). All other biomarker levels did not differ significantly between patients and controls (eTable 1).

### PICU patients with and without delirium

Tau protein at day 3 (15.3 [10.4, 25.1] pg/ml vs. 10.4 [5.2, 13.4] pg/ml, *p* = 0.019), as well as UCHL-1 at day 3 (25.2 [20.2, 35.7] pg/ml vs. 11.2 [9.3, 21.3] pg/ml, *p* = 0.034) and day 5 (20.7 [16.5, 27.2] pg/ml vs. 9.1 [6.8, 17.0] pg/ml, *p* = 0.023) were significantly higher in patients with delirium compared to non-delirious children. After adjustment for age, these significant differences disappeared. All other blood-based biomarkers showed similar levels in both groups (eTable 2).

No statistically significant differences were observed regarding CSF biomarker comparisons (eTable 3).

Mixed linear effect models revealed no significant trajectories between biomarker levels at the different time points and group comparisons.

The global test for differences between the qEEG model and intercept-only model was significant (χ2(14) = 39.3, *p* < 0.01). Yet, no single qEEG parameter significantly differed between delirious and non-delirious patients.

### Patients with ME and sepsis

Inflammatory biomarkers CRP and PCT were significantly elevated in patients with sepsis compared to patients with ME at nearly all study days (eTable 1).

**Fig. 2:**
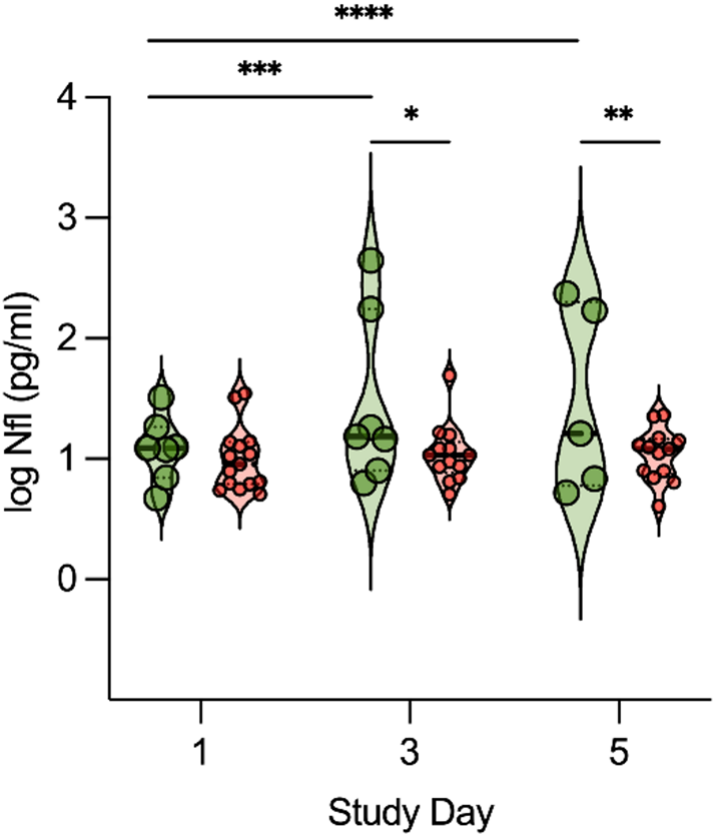
Comparison of neurofilament light chain (NfL) levels over time between patients with sepsis (orange violinplot) and meningoencephalitis (green violinplot). *p = 0.011, **p = 0.005, ***p = 0.012, ****p < 0.001.

NfL concentrations were significantly higher in patients with ME at day 3 (16.8 [12.9, 77.5] pg/ml vs. 10.7 [8.6, 12.2] pg/ml, *p* = 0.011) and day 5 (16.2 [6.8, 170.4] pg/ml vs. 12.0 [7.9, 14.3] pg/ml, *p* = 0.005) compared to patients with sepsis (Fig. 2). In contrast to sepsis patients, NfL levels increased also significantly over time in patients with ME, even after correction for age (Fig. 2). All other blood-based biomarkers had comparable levels between both groups (eTable 1).

In CSF samples (eTable 3), only tau protein levels were higher in patients with sepsis (401.9 [243.4, 1461.6] pg/ml vs. 127.1 [97.6, 169.8] pg/ml, *p* = 0.013), but missed statistical significance after correction for age (*p* = 0.202).

### PICU patients with and without abnormal EEG

Blood and CSF biomarker levels were mostly similar between patients with normal (*n* = 16) and abnormal EEG (*n* = 8) (eTable 2, eTable 3). Only UCHL-1 at day 3 was higher in patients with abnormal EEG when corrected for age (25.8 [14.6, 34.9] ng/ml vs. 18.7 [10.6, 26.8] ng/ml, *p* = 0.026).

Mixed linear effect models revealed no significant trajectories between biomarker levels at the different time points and group comparisons.

Exploratory analyses of qEEG parameters associated with encephalopathy revealed significant correlations with fluid biomarkers across age groups. The analyses focused on NfL, Tau, and UCHL-1, as these biomarkers exhibited clear changes over time. A detailed summary of the results is presented in Table 3. UCHL-1 and Tau showed significant positive correlations with the power of slow-wave activity in the delta band, while negative correlations were observed with the power of fast activity. Consistently, peak frequency was lower in the 1–8-year age group for higher UCHL-1 values. Connectivity measures predominantly correlated with fluid biomarkers of neuroinflammation and neuroaxonal damage in children under 1 year of age. These correlations indicated a positive association with excessive slow-wave phase coupling and elevated amplitude envelope fluctuations across all frequencies, indicating brain dysfunction.

**Table 3:** Summary of quantitative EEG parameter correlations With body-fluid biomarkers. Pearson correlation coefficients are displayed. With significant values highlighted using bold font. AECc = amplitude envelope correlation. PLI = phase lag index. Pow = power. Peak freq = peak frequency.

	AECc delta	AECc theta	AECc alpha	AECc beta1	PLI delta	PLI theta	PLI alpha	PLI beta	Pow delta	Pow theta	Pow alpha	Pow beta	Peak freq
Age Group: 1–11 months													
NfL max	0.30	-0.03	-0.16	-0.25	-0.32	-0.15	-0.24	-0.18	-0.24	-0.07	-0.19	-0.04	-0.26
NfL mean	0.23	-0.11	-0.23	-0.29	-0.38	-0.19	-0.26	-0.20	-0.32	-0.07	-0.15	0.00	-0.28
Tau max	0.22	0.48	**0.59**	**0.60**	0.26	0.26	0.20	-0.04	**0.38**	-0.27	**-0.57**	**-0.62**	-0.14
Tau mean	0.31	**0.47**	**0.54**	**0.57**	**0.31**	**0.36**	0.23	-0.04	**0.42**	-0.21	**-0.60**	**-0.65**	-0.13
UCHL-1 max	0.32	**0.47**	**0.49**	0.58	0.36	0.38	0.29	0.08	0.46	-0.29	**-0.34**	**-0.48**	0.14
UCHL-1 mean	0.48	**0.53**	**0.49**	**0.48**	0.21	0.29	0.17	0.00	**0.36**	-0.23	-0.44	**-0.52**	0.00
**Age Group: 1–8 years**													
NfL max	0.08	0.32	0.28	0.27	0.02	0.01	-0.22	0.06	0.05	-0.12	-0.16	-0.41	-0.38
NfL mean	0.07	0.34	0.29	0.28	0.03	0.04	-0.25	0.07	0.03	-0.08	-0.19	-0.44	-0.44
Tau max	-0.32	-0.27	-0.20	-0.19	-0.25	0.26	0.24	-0.44	0.06	0.19	-0.21	-0.22	0.06
Tau mean	-0.30	-0.21	-0.13	-0.13	-0.20	0.29	0.21	-0.43	0.05	0.22	-0.24	-0.28	0.02
UCHL-1 max	-0.22	0.08	0.02	-0.02	0.04	0.09	-0.43	-0.07	**0.38**	-0.20	**-0.59**	**-0.44**	**-0.37**
UCHL-1 mean	-0.23	0.08	0.05	0.00	0.07	0.12	-0.40	-0.14	**0.44**	-0.24	**-0.65**	-0.48	**-0.37**
**Age Group: 9–17 years**													
NfL max	-0.10	0.10	-0.17	0.05	0.01	-0.14	-0.11	-0.32	0.12	0.02	-0.17	-0.13	-0.20
NfL mean	-0.11	0.09	-0.18	0.02	-0.01	-0.14	-0.11	-0.34	0.12	0.02	-0.16	-0.12	-0.19
Tau max	-0.50	-0.61	-0.72	**-0.96**	**-0.88**	-0.43	-0.17	-0.63	-0.22	0.11	0.21	0.44	0.14
Tau mean	-0.52	-0.66	-0.73	**-0.96**	**-0.89**	-0.44	-0.19	-0.58	-0.24	0.13	0.21	0.47	0.14
UCHL-1 max	0.58	0.74	0.80	**0.94**	**0.89**	0.49	0.25	0.57	0.23	-0.18	-0.16	-0.47	-0.07
UCHL-1 mean	0.65	0.79	0.88	**0.90**	**0.89**	0.55	0.33	0.59	0.20	-0.23	-0.10	-0.45	0.02

### Functional and neurocognitive status before hospital admission and after three months

Patients with and without delirium, as well as with ME and sepsis were comparable regarding their functional and neurological (POPC, PCPC) status before hospital admission (Tables 1 and 2). Patients with delirium had a higher FSS (10.0 [9.8, 12.0] vs. 7.5 [7.0, 9.0], *p* = 0.009) and a significantly lower GCS at day 1 (13.5 [13.0, 14.0] vs. 15.0 [15.0, 15.0], *p* < 0.001). Similar results were observed by comparing patients with ME and sepsis, with the sepsis cohort presenting with a higher FSS (10.0 [8.5, 11.5] vs. 7.0 [7.0, 9.0], *p* = 0.019) and a lower GCS (14.0 [13.0, 15.0] vs. 15.0 [15.0, 15.0], *p* = 0.045) only at day 1. No differences were observed in patients with and without EEG abnormalities. No severe neurological complications were observed during the study, regardless of group classification. All groups were comparable in their functional (FSS) and neurological (GCS, RASS, PCPC, POPC) status after three months (Table 2).

## Discussion

In the present study, delirium was not associated with increased morbidity or mortality among PICU patients with acute infectious diseases. The overall delirium prevalence was 50%, which is comparable to recent data^10,11^. Interestingly, these results contrast the delirium prevalence of only 4% in SARS-CoV-2-infected children, suggesting a stronger inflammatory stimulus by ME or sepsis compared to SARS-CoV-2. ^25^ Despite slight differences in functional and neurological measures within the acute phase, the outcome was comparable between children with and without delirium after three months. In accordance with the clinical follow-up, biomarker levels of brain injury in blood and CSF were elevated compared to healthy controls, but similar between patients with and without delirium. No clinically relevant correlations between blood biomarkers and outcome parameters were observed. Our data confirm that delirium is frequent in pediatric patients and reflects a transient neurological dysfunction that is rather unlikely to persist as subsequent neurological impairment.

Biomarker levels of neuronal injury (NSE) were elevated in children after cardiac surgery, but with only moderate correlation between biomarker concentrations and delirium scores^26,27^. On the other hand, similar concentrations of IL-6 in children with influenza and either delirium or febrile seizures have been reported, indicating a mismatch between biomarker levels and the clinical status^28^. In this context, no association of elevated CRP and PCT values and delirium in septic children was observed^29^. Furthermore, levels of NSE and S100B were similarly elevated in children with and without sepsis-associated encephalopathy and full neurological recovery after three months, indicating preserved CNS integrity at the cellular level^30^.

Neurofilament light chains were extensively evaluated over the last decades and correlate with delirium severity in adults. ^2,7,31^ However, in children NfL levels remain normal, even in the presence of neurological symptoms^34^. In contrast, we observed significantly elevated NfL blood levels in children with ME, but without acute or persistent neurological impairments, which may reflect the potential of the pediatric brain to compensate for CNS affection to a certain degree.

Normal and abnormal EEG findings from intermittent EEG recordings did not correlate with changes in serum and CSF biomarker levels. Thus, intermittent EEG seems unfavorable for outcome prediction here. Exploratory qEEG patterns in pediatric delirium differed significantly compared to adult data, possibly reflecting discrepancies between the phenotypical and neurophysiological characteristics of delirium^35,36^. Increased delta power emerges as the most prominent finding in qEEG analyses of delirium, showing significant positive correlations with UCHL-1 and Tau protein, which indicates that encephalopathy severity corresponds to the extent of neuronal injury and neuroinflammation. Additionally, UCHL-1 showed strong positive correlations with AECc in the delta band, supporting the concept of excessive network inhibition^33^. Decreased peak frequency was also significantly associated with elevated Tau protein levels, aligning with established patterns in encephalopathy^38^. In summary, our results support an advanced characterization of delirium endotypes (e.g. using qEEG) beyond investigations of the binary phenotype (i.e. being delirious or not)^39^.

### Strengths and limitations

The broad panel of eleven blood-based and five CSF biomarkers allowed us to assess a widespread range of compartments, including neuronal, axonal, glial, vascular as well as to quantify the impact of systemic infection and primary CNS infection. Although the sample size was small, patients underwent repeated comprehensive assessments with neuropediatric examinations, EEG as well as blood and CSF sampling for biomarker analysis. This multimodal diagnostic approach at different time points allowed us to assess different qualities of neurological impairment and to quantify the extent of neurological injury at the cellular level. Unfortunately, we were not able to perform routine magnetic resonance imaging (MRI), which would have enabled us to assess brain injury at an imaging level. Only three PICU patients were examined by MRI without pathological results. Due to the COVID pandemia and legal restrictions of hospital visits it was impossible to assess all pediatric patients in our outpatient department. Instead of in-house examination, we performed standardized telephone interviews in these children, which allowed for reliable neurological outcome assessment, but only to a certain degree for an evaluation of neurodevelopmental delays. A larger sample size and comprehensive long-term neurocognitive testing should be part of future studies to verify the results of the present study. Furthermore, a control group without infection is warranted to assess the clinical utility of blood-based biomarker results over the time course of PICU patients.

## Conclusions

Pediatric delirium is unlikely to result in sustained brain injury and subsequent neurological impairment, despite transient brain dysfunction and corresponding transient elevations of biomarkers in specific subgroups. ME as a primary CNS infection and sepsis with secondary brain involvement trigger delirium to a comparable extent. Future studies might also focus on even more severely ill children to validate our observations. Intermittent or continuous qEEG monitoring might represent a clinically useful instrument to assess delirium in the future.

## Supplementary Information

Below is the link to the electronic supplementary material.

Supplementary Material 1

Supplementary Material 2

Supplementary Material 3

## Data Availability

The data underlying this article are available in the article and in its online supplementary material. Request for additional information can be made to the corresponding author.

## Abbreviations

AEC: amplitude envelope correlation
AECc: corrected amplitude envelope correlation
CNS: central nervous system
CRP: C-reactive protein
CSF: cerebrospinal fluid
CV: coefficient of variation
ECLIA: electrochemiluminescence-based immunoassays
EEG: electroencephalogramm
ELISA: enzyme-linked immunosorbent assays
ET 6-6-R: Entwicklungstest für Kinder von 6 Monaten bis 6 Jahren
FSS: Functional Status Scale
GCS: Glasgow Coma Scale
GFAP: glial fibrillary acidic protein
IL-6: Interleukin-6
KABC-II: Kaufman Assessment Battery for Children
LMM: linear mixed model
ME: meningoencephalitis
MFED: Münchener Funktionelle Entwicklungsdiagnostik
MRI: magnetic resonance imaging
NfH: neurofilament heavy chains
NfL: neurofilament light chains
NSE: neuro-specific enolase
NT-proCNP: N-Terminal pro C-type natriuretic peptide
pCAM: pediatric Confusion Assessment Method
PCPC: pediatric cerebral performance scale
PCT: Procalcitonin
PICU: pediatric intensive care unit
PLI: phase lag index
POPC: pediatric overall performance category
psCAM-ICU: preschool CAM-ICU
pSOFA: pediatric Sequential Organ Failure Assessment
qEEG: quantitative electroencephalogramm
RASS: Richmond Agitation and Sedation Scale
S100B: S100 calcium-binding protein B
SOS-PD: Sophia Observation Withdrawal Symptoms-scale for pediatric delirium
UCHL-1: Ubiquitin carboxy-terminal hydrolase L1
