# Supplementary material for: Impact of meningoencephalitis and sepsis on delirium and subsequent neurological impairment in pediatric patients: a prospective proof-of-concept biomarker and EEG study
Source: Sci Rep. 2025 Dec 8;15:43492. doi: 10.1038/s41598-025-31058-2 (PMC12695873; doi:10.1038/s41598-025-31058-2)
Supplement: Supplementary file 1 — Supplementary Material 1 [file 41598_2025_31058_MOESM1_ESM.docx]

|  | **Patients** | **Controls** | ***p value*** | ***p value  (age-corrected)*** | **ME** | **Sepsis** | ***p value*** | ***p value (age-corrected)*** |
| --- | --- | --- | --- | --- | --- | --- | --- | --- |
|  |  |  |  |  |  |  |  |  |
| **Number (n)** | **24** | **11** |  |  | **9** | **15** |  |  |
| **WBC (10^9/l) day 1** | 12.6 [6.3, 23.1] | 5.8 [5.0, 7.6] | **<0.001** | **<0.001** | 6.4 [5.4, 7.6] | 18.5 [13.2, 25.2] | **<0.001** | **<0.001** |
| **WBC (10^9/l) day 3** | 7.4 [5.5, 12.6] | NA | NA | NA | 5.2 [4.6, 6.2] | 12.5 [7.0, 13.6] | **0.003** | **0.006** |
| **WBC (10^9/l) day 5** | 8.1 [5.8, 10.6] | NA | NA | NA | 5.7 [4.6, 5.8] | 9.6 [8.0, 11.5] | **0.009** | **0.019** |
| **CRP (mg/dl) day 1** | 39.7 [1.5, 156.0] | 0.0 [0.0, 0.5] | **<0.001** | **<0.001** | 0.0 [0.0, 1.6] | 114.0 [43.8, 208.0] | **<0.001** | **<0.001** |
| **CRP (mg/dl) day 3** | 17.9 [1.2, 150.5] | NA | NA | NA | 1.0 [0.0, 1.3] | 134.0 [20.2, 212.0] | **<0.001** | **<0.001** |
| **CRP (mg/dl) day 5** | 6.4 [0.8, 74.2] | NA | NA | NA | 0.0 [0.0, 0.0] | 46.0 [5.7, 87.7] | **<0.001** | **<0.001** |
| **PCT (ng/ml) day 1** | 0.8 [0.1, 16.7] | 0.1 [0.0, 0.1] | **0.003** | **0.003** | 0.1 [0.0, 0.1] | 5.9 [0.8, 35.4] | **<0.001** | **<0.001** |
| **PCT (ng/ml) day 3** | 4.8 [0.2, 10.5] | NA | NA | NA | 0.1 [0.1, 0.1] | 6.4 [3.1, 14.3] | **<0.001** | **<0.001** |
| **PCT (ng/ml) day 5** | 1.3 [0.1, 2.4] | NA | NA | NA | 0.1 [0.1, 0.1] | 2.0 [0.5, 3.1] | 0.070 | **0.009** |
| **IL-6 (pg/ml) day 1** | 53.1 [11.0, 201.0] | 1.0 [1.0, 2.0] | **<0.001** | **<0.001** | 6.4 [1.9, 17.2] | 95.0 [50.7, 253.0] | **<0.001** | **<0.001** |
| **IL-6 (pg/ml) day 3** | 10.7 [3.0, 31.9] | NA | NA | NA | 5.1 [0.8, 9.8] | 16.5 [4.0, 74.2] | 0.033 | **0.034** |
| **IL-6 (pg/ml) day 5** | 3.6 [2.1, 8.9] | NA | NA | NA | 3.6 [1.0, 4.0] | 3.6 [2.4, 21.3] | 0.431 | 0.431 |
| **NSE day 1 (pg/ml)** | 781.2 [529.2, 991.7] | 1760.4 [1216.5, 2550.7] | **<0.001** | **<0.001** | 611.4 [495.0, 822.3] | 885.0 [612.3, 1029.7] | 0.669 | 0.900 |
| **NSE day 3 (pg/ml)** | 1050.0 [735.2, 1402.0] | NA | NA | NA | 1204.7 [738.2, 1543.8] | 1009.7 [752.4, 1323.7] | 0.835 | 0.605 |
| **NSE day 5 (pg/ml)** | 1045.1 [792.4, 1652.0] | NA | NA | NA | 805.2 [537.9, 1931.2] | 1093.8 [972.9, 1531.5] | 0.617 | 0.887 |
| **GFAP day 1 (pg/ml)** | 194.8 [156.0, 362.4] | 233.2 [164.9, 294.1] | 0.959 | 0.778 | 159.8 [107.5, 263.5] | 214.3 [175.8, 362.4] | 0.481 | 0.999 |
| **GFAP day 3 (pg/ml)** | 135.6 [106.2, 261.1] | NA | NA | NA | 118.1 [90.7, 219.3] | 180.4 [116.1, 261.1] | 0.639 | 0.962 |
| **GFAP day 5 (pg/ml)** | 115.1 [97.2, 222.7] | NA | NA | NA | 95.2 [55.3, 107.0] | 149.2 [110.1, 322.2] | 0.300 | 0.843 |
| **NfH day 1 (ng/ml)** | 1.1 [0.2, 4.0] | 0.9 [0.2, 1.4] | 0.954 | 0.912 | 4.7 [3.4, 8.6] | 0.7 [0.2, 1.8] | 0.814 | 0.669 |
| **NfH day 3 (ng/ml)** | 1.5 [0.5, 3.7] | NA | NA | NA | 4.3 [2.8, 14.8] | 1.2 [0.5, 1.7] | 0.238 | 0.143 |
| **NfH day 5 (ng/ml)** | 1.1 [0.7, 4.5] | NA | NA | NA | 10.9 [5.5, 16.9] | 1.0 [0.8, 2.9] | 0.132 | 0.073 |
| **NfL day 1 (pg/ml)** | 11.0 [6.4, 13.9] | 7.9 [5.3, 10.5] | 0.221 | 0.219 | 12.4 [10.7, 20.3] | 9.0 [6.2, 13.3] | 0.678 | 0.670 |
| **NfL day 3 (pg/ml)** | 12.1 [8.6, 16.6] | NA | NA | NA | 16.8 [12.9, 77.5] | 10.7 [8.6, 12.2] | **0.009** | **0.011** |
| **NfL day 5 (pg/ml)** | 12.3 [7.2, 15.8] | NA | NA | NA | 16.2 [6.8, 170.4] | 12.0 [7.9, 14.3] | **0.004** | **0.005** |
| **NT-proCNP day 1 (pmol/l)** | 74.2 [51.3, 118.2] | 63.1 [61.7, 85.7] | 0.993 | 0.959 | 91.2 [52.4, 112.1] | 72.4 [50.7, 139.3] | 0.991 | 0.574 |
| **NT-proCNP day 3 (pmol/l)** | 78.1 [54.1, 135.2] | NA | NA | NA | 100.9 [68.5, 120.6] | 61.9 [43.4, 138.1] | 0.805 | 0.177 |
| **NT-proCNP day 5 (pmol/l)** | 82.7 [55.7, 122.3] | NA | NA | NA | 75.8 [61.4, 103.8] | 82.7 [43.5, 123.5] | 0.684 | 0.120 |
| **S100B day 1 (ng/ml)** | 7.9 [3.5, 18.0] | 7.9 [6.0, 10.4] | 0.871 | 0.881 | 13.0 [8.0, 21.8] | 5.2 [2.8, 11.3] | 0.121 | 0.202 |
| **S100B day 3 (ng/ml)** | 8.3 [3.3, 12.7] | NA | NA | NA | 11.5 [7.2, 13.8] | 7.0 [3.1, 11.4] | 0.999 | 0.978 |
| **S100B day 5 (ng/ml)** | 8.8 [3.4, 11.9] | NA | NA | NA | 11.1 [9.6, 18.0] | 4.7 [2.8, 10.9] | 0.131 | 0.224 |
| **Tau day 1 (ng/ml)** | 9.1 [8.0, 17.8] | 11.0 [7.8, 12.7] | 0.206 | 0.167 | 8.4 [8.0, 11.1] | 14.2 [8.7, 24.1] | 0.378 | 0.858 |
| **Tau day 3 (ng/ml)** | 12.4 [7.1, 18.2] | NA | NA | NA | 8.3 [5.3, 14.2] | 12.6 [10.4, 18.2] | 0.297 | 0.740 |
| **Tau day 5 (ng/ml)** | 9.9 [8.9, 11.8] | NA | NA | NA | 9.7 [9.0, 10.0] | 11.5 [8.8, 12.1] | 0.979 | 0.450 |
| **UCHL1 day 1 (ng/ml)** | 22.2 [14.9, 37.8] | 14.2 [6.0, 20.7] | 0.059 | **0.032** | 14.9 [9.6, 19.0] | 26.4 [18.9, 47.7] | 0.246 | 0.686 |
| **UCHL1 day 3 (ng/ml)** | 19.4 [10.8, 27.7] | NA | NA | NA | 14.6 [10.7, 22.0] | 24.3 [11.2, 27.7] | 0.736 | 0.983 |
| **UCHL1 day 5 (ng/ml)** | 16.2 [8.6, 22.7] | NA | NA | NA | 14.7 [8.1, 16.8] | 18.5 [10.1, 27.0] | 0.991 | 0.634 |

eTable 1: Blood-based biomarker results of pediatric patients.

CRP=C-reactive protein; GFAP=Glial fibrillary acidic protein; IL-6=Interleukin-6; ME=Meningoencephalitis; NA=Not accessible; NfL=Neurofilament light chain; NfH=Neurofilament heavy chain; NT-proCNP= N-Terminal pro C-type natriuretic peptide; NSE=Neuron-specific enolase; PCT=Procalcitonin; S100B=S100 calcium-binding protein B; UCHL-1= Ubiquitin carboxy-terminal hydrolase L1; WBC= WBC=White blood count. Values are given as medians [interquartile range]
