# Supplementary material for: Impact of meningoencephalitis and sepsis on delirium and subsequent neurological impairment in pediatric patients: a prospective proof-of-concept biomarker and EEG study
Source: Sci Rep. 2025 Dec 8;15:43492. doi: 10.1038/s41598-025-31058-2 (PMC12695873; doi:10.1038/s41598-025-31058-2)
Supplement: Supplementary file 2 — Supplementary Material 2 [file 41598_2025_31058_MOESM2_ESM.docx]

|  | **Delirium** | **No delirium** | ***p value*** | ***p value (age-corrected)*** | **Abnormal EEG** | **Normal EEG** | ***p value*** | ***p value (age-corrected)*** |
| --- | --- | --- | --- | --- | --- | --- | --- | --- |
|  |  |  |  |  |  |  |  |  |
| **Number (n)** | **12** | **12** |  |  | **8** | **16** |  |  |
| **WBC (10^9/l) day 1** | 13.8 [8.6, 24.4] | 7.4 [6.0, 19.5] | 0.270 | 0.447 | 8.2 [5.9, 17.1] | 14.4 [7.0, 23.1] | 0.453 | 0.614 |
| **WBC (10^9/l) day 3** | 8.4 [7.0, 13.6] | 5.7 [4.8, 12.5] | 0.216 | 0.368 | 8.7 [5.8, 13.2] | 7.3 [5.5, 12.6] | 0.638 | 0.480 |
| **WBC (10^9/l) day 5** | 8.5 [6.5, 10.0] | 6.4 [5.6, 10.9] | 0.382 | 0.584 | 8.6 [7.3, 11.4] | 6.4 [5.8, 10.2] | 0.368 | 0.264 |
| **CRP (mg/dl) day 1** | 43.8 [8.3, 156.0] | 29.0 [0.0, 123.8] | 0.346 | 0.565 | 78.9 [8.2, 239.2] | 29.0 [1.5, 112.0] | 0.533 | 0.360 |
| **CRP (mg/dl) day 3** | 50.6 [8.0, 201.5] | 10.1 [0.0, 134.2] | 0.232 | 0.415 | 139.2 [7.6, 210.5] | 14.4 [1.2, 112.2] | 0.272 | 0.168 |
| **CRP (mg/dl) day 5** | 9.2 [3.2, 87.7] | 5.8 [0.3, 50.5] | 0.487 | 0.730 | 42.4 [2.6, 96.6] | 4.5 [0.0, 41.4] | 0.235 | 0.144 |
| **PCT (ng/ml) day 1** | 0.8 [0.3, 14.2] | 2.3 [0.1, 15.2] | 0.759 | 0.923 | 0.7 [0.3, 62.1] | 2.3 [0.1, 10.2] | 0.413 | 0.469 |
| **PCT (ng/ml) day 3** | 5.3 [0.2, 13.4] | 3.7 [0.2, 7.1] | 0.442 | 0.362 | 9.9 [0.2, 16.4] | 4.2 [0.1, 6.4] | 0.670 | 0.722 |
| **PCT (ng/ml) day 5** | 1.6 [0.1, 2.9] | 0.3 [0.1, 2.0] | 0.619 | 0.508 | 2.0 [0.1, 3.0] | 0.8 [0.1, 2.0] | 0.745 | 0.790 |
| **IL-6 (pg/ml) day 1** | 50.7 [20.1, 154.2] | 62.6 [7.4, 195.5] | 0.334 | 0.410 | 44.7 [16.3, 231.8] | 62.6 [13.0, 111.5] | 0.855 | 0.992 |
| **IL-6 (pg/ml) day 3** | 8.8 [4.6, 41.3] | 16.5 [0.0, 18.6] | 0.251 | 0.305 | 69.4 [5.3, 97.5] | 8.8 [2.9, 17.7] | 0.204 | 0.166 |
| **IL-6 (pg/ml) day 5** | 3.8 [2.4, 24.7] | 3.6 [1.9, 5.8] | 0.470 | 0.557 | 8.9 [4.0, 27.8] | 3.3 [1.7, 3.8] | 0.273 | 0.237 |
| **NSE day 1 (pg/ml)** | 966.8 [640.3, 1184.0] | 696.3 [430.5, 856.2] | 0.129 | 0.298 | 812.1 [558.9, 932.8] | 744.3 [546.3, 990.4] | 0.934 | 0.697 |
| **NSE day 3 (pg/ml)** | 926.0 [721.5, 1332.4] | 1235.8 [798.6, 1402.0] | 0.966 | 0.542 | 1201.1 [826.4, 1650.0] | 1050.0 [721.5, 1271.9] | 0.489 | 0.264 |
| **NSE day 5 (pg/ml)** | 1359.9 [995.7, 2196.2] | 925.8 [604.8, 1097.5] | 0.109 | 0.279 | 1045.1 [869.8, 1529.6] | 1048.0 [792.4, 2112.5] | 0.626 | 0.883 |
| **GFAP day 1 (pg/ml)** | 225.9 [168.3, 444.2] | 188.5 [107.5, 231.8] | 0.131 | 0.768 | 186.4 [98.6, 233.8] | 202.0 [159.8, 462.5] | 0.161 | 0.582 |
| **GFAP day 3 (pg/ml)** | 189.8 [136.1, 494.4] | 106.2 [75.7, 161.9] | 0.067 | 0.898 | 97.3 [73.4, 188.6] | 158.9 [128.0, 402.5] | 0.270 | 0.870 |
| **GFAP day 5 (pg/ml)** | 169.9 [117.6, 329.5] | 105.0 [56.4, 120.9] | 0.057 | 0.794 | 107.0 [56.6, 129.6] | 124.7 [106.3, 292.5] | 0.189 | 0.655 |
| **NfH day 1 (ng/ml)** | 0.7 [0.2, 1.8] | 2.5 [0.3, 4.1] | 0.870 | 0.959 | 0.7 [0.0, 3.3] | 1.1 [0.3, 4.3] | 0.538 | 0.640 |
| **NfH day 3 (ng/ml)** | 1.3 [0.5, 2.5] | 1.7 [0.4, 4.1] | 0.848 | 0.982 | 1.6 [0.5, 1.7] | 1.3 [0.4, 4.0] | 0.309 | 0.389 |
| **NfH day 5 (ng/ml)** | 1.2 [1.0, 5.7] | 1.1 [0.5, 3.4] | 0.809 | 0.984 | 2.4 [1.0, 4.5] | 1.0 [0.4, 3.7] | 0.847 | 0.787 |
| **NfL day 1 (pg/ml)** | 10.6 [5.9, 13.4] | 12.2 [7.7, 15.0] | 0.743 | 0.799 | 11.7 [9.5, 15.0] | 9.0 [6.3, 13.4] | 0.734 | 0.932 |
| **NfL day 3 (pg/ml)** | 10.7 [8.6, 14.9] | 14.5 [8.8, 17.4] | 0.945 | 0.545 | 15.3 [12.1, 32.9] | 10.1 [8.1, 15.4] | 0.468 | 0.647 |
| **NfL day 5 (pg/ml)** | 12.5 [7.6, 14.4] | 11.9 [7.2, 20.9] | 0.443 | 0.869 | 14.9 [10.3, 96.5] | 11.2 [6.9, 13.7] | 0.074 | 0.136 |
| **NT-proCNP day 1 (pmol/l)** | 59.1 [45.5, 116.5] | 99.2 [54.7, 118.2] | 0.541 | 0.148 | 88.5 [52.8, 118.2] | 71.1 [47.2, 118.8] | 0.672 | 0.417 |
| **NT-proCNP day 3 (pmol/l)** | 63.2 [43.4, 136.8] | 100.6 [60.5, 124.2] | 0.424 | 0.104 | 78.1 [56.1, 136.5] | 84.4 [54.1, 124.3] | 0.854 | 0.849 |
| **NT-proCNP day 5 (pmol/l)** | 82.7 [57.9, 121.0] | 79.6 [54.9, 120.2] | 0.495 | 0.135 | 75.9 [38.4, 134.1] | 82.7 [59.8, 119.9] | 0.640 | 0.910 |
| **S100B day 1 (ng/ml)** | 6.2 [4.7, 18.9] | 11.3 [3.0, 14.7] | 0.872 | 0.515 | 9.8 [4.7, 20.2] | 7.9 [2.2, 13.4] | 0.537 | 0.684 |
| **S100B day 3 (ng/ml)** | 8.8 [4.7, 15.0] | 7.7 [2.8, 11.6] | 0.340 | 0.160 | 10.2 [5.3, 13.0] | 8.2 [2.8, 12.7] | 0.477 | 0.617 |
| **S100B day 5 (ng/ml)** | 7.9 [4.1, 10.9] | 9.9 [2.8, 12.5] | 0.611 | 0.324 | 4.7 [2.9, 10.5] | 10.6 [6.4, 11.9] | 0.176 | 0.129 |
| **Tau day 1 (ng/ml)** | 16.1 [9.0, 30.4] | 8.7 [8.0, 14.2] | 0.059 | 0.623 | 11.6 [8.0, 21.2] | 9.1 [8.2, 17.8] | 0.827 | 0.167 |
| **Tau day 3 (ng/ml)** | 15.3 [10.4, 25.1] | 10.4 [5.2, 13.4] | **0.019** | 0.323 | 10.4 [4.4, 18.5] | 12.5 [10.2, 17.2] | 0.581 | 0.678 |
| **Tau day 5 (ng/ml)** | 10.8 [9.0, 13.6] | 9.5 [8.5, 11.6] | 0.617 | 0.388 | 9.2 [8.9, 11.7] | 10.0 [8.7, 11.8] | 0.680 | 0.578 |
| **UCHL-1 day 1 (ng/ml)** | 26.4 [17.2, 45.1] | 17.5 [9.0, 26.2] | 0.056 | 0.533 | 35.7 [12.7, 47.7] | 21.1 [15.9, 25.2] | 0.712 | 0.093 |
| **UCHL-1 day 3 (ng/ml)** | 25.2 [20.2, 35.7] | 11.2 [9.3, 21.3] | **0.034** | 0.399 | 25.8 [14.6, 34.9] | 18.7 [10.6, 26.8] | 0.365 | **0.026** |
| **UCHL-1 day 5 (ng/ml)** | 20.7 [16.5, 27.2] | 9.1 [6.8, 17.0] | **0.023** | 0.282 | 17.7 [10.7, 23.2] | 15.6 [9.1, 22.4] | 0.627 | 0.503 |

eTable 2: Blood-based biomarker results in pediatric patients with and without delirium as well as normal and abnormal electroencephalography findings.

CRP=C-reactive protein; EEG=Electroencephalography; GFAP=Glial fibrillary acidic protein; IL-6=Interleukin-6; NfL=Neurofilament light chain; NfH=Neurofilament heavy chain; NT-proCNP= N-Terminal pro C-type natriuretic peptide; NSE=Neuron-specific enolase; PCT=Procalcitonin; S100B=S100 calcium-binding protein B; UCHL-1= Ubiquitin carboxy-terminal hydrolase L1; WBC= WBC=White blood count. Values are given as medians [interquartile range]
