# Supplementary material for: Impact of meningoencephalitis and sepsis on delirium and subsequent neurological impairment in pediatric patients: a prospective proof-of-concept biomarker and EEG study
Source: Sci Rep. 2025 Dec 8;15:43492. doi: 10.1038/s41598-025-31058-2 (PMC12695873; doi:10.1038/s41598-025-31058-2)
Supplement: Supplementary file 3 — Supplementary Material 3 [file 41598_2025_31058_MOESM3_ESM.docx]

|  | **ME** | **Sepsis** | ***p value*** | ***p value  (age-corrected)*** | **Delirium** | **No delirium** | ***p value*** | ***p value (age-corrected)*** | **Abnormal EEG** | **Normal EEG** | ***p value*** | ***p value (age-corrected)*** |
| --- | --- | --- | --- | --- | --- | --- | --- | --- | --- | --- | --- | --- |
|  |  |  |  |  |  |  |  |  |  |  |  |  |
| **Number (n)** | 9 | 15 |  |  | 12 | 12 |  |  | 8 | 16 |  |  |
| **CSF WBC (10^9)** | 75.0 [31.0, 213.0] | 2.5 [1.2,3.0] | **0.002** | 0.062 | 9.0 [2.0,295.0] | 24.0 [2.8,74.2] | 0.800 | 0.315 | 31.0 [17.0,53.0] | 13.0 [1.8,168.0] | 0.976 | 0.674 |
| **CSF lymphocytes (%)** | 89.0 [86.0, 95.0] | 52.5 [25.5,74.5] | 0.742 | 0.549 | 71.0 [27.0,85.0] | 88.5 [73.0,93.5] | 0.170 | 0.222 | 89.0 [80.0,94.5] | 85.5 [28.8,91.8] | 0.368 | 0.396 |
| **CSF neutrophils (%)** | 1.0 [0.0, 17.8] | 5.0 [0.0,23.8] | 0.664 | 0.196 | 10.0 [0.0,65.0] | 0.0 [0.0,2.0] | 0.260 | 0.496 | 2.0 [1.0,6.0] | 0.0 [0.0,65.0] | 0.262 | 0.214 |
| **CSF protein total (mg/dl)** | 450.0 [383.0, 878.0] | 569.0 [260.0,658.0] | 0.501 | 0.935 | 664.0 [346.0,826.0] | 444.0 [380.8,503.0] | 0.553 | 0.418 | 438.0 [410.5,548.0] | 481.0 [317.0,774.0] | 0.867 | 0.750 |
| **CSF albumin (mg/l)** | 270.0 [230.0, 615.0] | 230.0 [117.5,340.0] | 0.134 | 0.582 | 400.0 [282.5,595.0] | 240.0 [220.0,305.0] | 0.509 | 0.518 | 280.0 [250.0,310.0] | 270.0 [220.0,460.0] | 0.851 | 0.985 |
| **Serum albumin (g/l)** | 40.0 [38.0, 42.4] | 28.3 [28.0,32.1] | **0.003** | **0.017** | 37.3 [34.9,37.8] | 40.6 [36.9,43.0] | 0.433 | 0.274 | 36.4 [32.3,40.4] | 38.0 [36.8,40.3] | 0.699 | 0.824 |
| **CSF / Serum albumin ratio** | 9.3 [5.7, 23.0] | 7.9 [3.7,12.1] | 0.332 | 0.910 | 12.0 [9.9,15.7] | 6.0 [5.1,10.9] | 0.897 | 0.768 | 8.5 [6.7,10.3] | 9.3 [5.0,15.9] | 0.671 | 0.779 |
| **CSF glucose (mmol/l)** | 3.2 [2.9, 3.5] | 3.9 [3.1,4.0] | 0.352 | 0.848 | 4.0 [3.1,4.0] | 3.2 [3.0,3.3] | 0.157 | 0.217 | 2.9 [2.9,3.0] | 3.5 [3.1,4.0] | 0.220 | 0.275 |
| **CSF lactate (mmol/l)** | 1.7 [1.5, 2.0] | 1.3 [1.3,1.7] | 0.627 | 0.958 | 2.2 [1.4,2.9] | 1.5 [1.3,1.7] | 0.064 | **0.046** | 1.3 [1.1,1.4] | 1.7 [1.4,2.3] | 0.079 | 0.065 |
| **CSF IgG (mg/l)** | 36.0 [20.0, 60.0] | 15.5 [8.0,27.5] | 0.109 | 0.595 | 44.0 [32.8,73.5] | 22.0 [17.5,29.5] | 0.362 | 0.250 | 29.0 [23.0,35.0] | 23.0 [18.0,47.0] | 0.933 | 0.890 |
| **Serum IgG (g/l)** | 7.9 [5.4, 9.5] | 5.7 [4.8,128.9] | 0.353 | 0.250 | 5.7 [5.2,7.8] | 7.9 [5.3,10.2] | 0.522 | 0.545 | 7.0 [6.4,7.6] | 7.6 [4.6,10.4] | 0.691 | 0.719 |
| **CSF / Serum IgG ratio** | 3.3 [2.9, 4.0] | 6.6 [6.3,6.9] | 0.465 | 0.179 | 11.3 [9.2,13.3] | 3.3 [2.9,4.0] | **0.026** | 0.053 | 4.6 [3.3,5.9] | 4.0 [3.3,6.0] | 0.656 | 0.755 |
| **CSF IgA (mg/l)** | 2.5 [1.7, 5.4] | 1.0 [0.8,1.1] | **0.025** | 0.264 | 2.5 [0.8,5.8] | 1.6 [1.3,2.1] | 0.880 | 0.806 | 1.3 [1.2,1.5] | 1.6 [1.1,4.1] | 0.559 | 0.630 |
| **Serum IgA (g/l)** | 0.7 [0.5, 0.9] | 0.2 [0.1,0.2] | 0.217 | 0.739 | 0.5 [0.5,0.6] | 0.5 [0.1,0.9] | 0.647 | 0.760 | 0.9 [0.9,0.9] | 0.4 [0.2,0.7] | 0.405 | 0.448 |
| **CSF / Serum IgA ratio** | 2.2 [1.9, 15.3] | 3.1 [2.5,5.3] | 0.724 | 0.661 | 7.5 [4.7,11.4] | 2.2 [1.9,3.1] | 0.468 | 0.510 | 4.7 [3.3,6.1] | 2.7 [2.0,12.3] | 0.865 | 0.873 |
| **CSF IgM (mg/l)** | 1.4 [0.8, 9.9] | 0.4 [0.4,0.5] | **0.048** | 0.249 | 3.4 [0.7,8.0] | 0.6 [0.5,1.2] | 0.380 | 0.306 | 0.7 [0.6,0.7] | 1.0 [0.5,6.1] | 0.535 | 0.617 |
| **Serum IgM (g/l)** | 0.8 [0.8, 1.5] | 0.5 [0.4,0.5] | **0.032** | 0.225 | 0.7 [0.6,1.2] | 0.8 [0.6,0.9] | 0.75 | 0.937 | 0.6 [0.6,0.7] | 0.8 [0.6,1.3] | 0.657 | 0.782 |
| **CSF / Serum IgM ratio** | 0.7 [0.6, 1.2] | 1.4 [0.9,1.6] | 0.855 | 0.560 | 1.4 [0.9,4.7] | 0.7 [0.6,1.2] | 0.39 | 0.419 | 1.0 [0.8,1.2] | 0.9 [0.6,1.7] | 0.798 | 0.917 |
| **CSF NfH (ng/ml)** | 5.5 [2.5, 9.9] | 2.7 [2.0,3.6] | 0.21 | 0.843 | 2.7 [2.2,4.6] | 7.2 [2.3,12.3] | 0.169 | 0.339 | 14.7 [8.7,16.6] | 2.6 [2.0,6.1] | 0.064 | 0.074 |
| **CSF NfL (pg/ml)** | 159.6 [121.7, 204.7] | 225.3 [196.3,274.6] | 0.639 | 0.946 | 249.9 [203.5,276.3] | 136.4 [117.0,186.7] | 0.099 | 0.123 | 246.9 [186.7,260.8] | 186.7 [114.8,218.1] | 0.733 | 0.607 |
| **CSF Tau (ng/ml)** | 127.1 [97.6, 169.8] | 401.9 [243.4,1461.6] | **0.013** | 0.202 | 322.7 [184.4,1196.7] | 138.8 [106.6,183.6] | 0.122 | 0.823 | 138.8 [127.1,1015.7] | 183.6 [114.5,362.3] | 0.634 | 0.144 |
| **CSF UCHL-1 (ng/ml)** | 862.9 [761.5, 1079.0] | 860.6 [734.5,1356.3] | 0.672 | 0.789 | 1068.1 [872.0,1324.7] | 805.7 [681.6,941.7] | 0.223 | 0.429 | 819.8 [812.8,1088.0] | 883.3 [655.2,1109.9] | 0.547 | 0.373 |
| **CSF GFAP (pg/ml)** | 4188.1 [3032.7, 4599.6] | 4677.6 [2436.7,10425.8] | 0.769 | 0.976 | 4638.6 [4599.6,8988.8] | 3071.3 [2808.1,4188.1] | 0.127 | 0.116 | 3071.3 [2885.3,6748.5] | 4467.0 [3198.2,4658.1] | 0.897 | 0.812 |

eTable 3: Cerebrospinal fluid-based biomarker results of pediatric patients.

CSF=Cerebrospinal fluid; EEG=Electroencephalography; GFAP=Glial fibrillary acidic protein; IgA=Immunoglobuline A; IgG=Immunoglobuline G; IgM=Immunoglobuline M; ME=Meningoencephalitis; NfH=Neurofilament heavy chain; NfL=Neurofilament light chain; UCHL-1=Ubiquitin carboxy-terminal hydrolase L1; WBC=White blood count. Values are given as medians [interquartile range]
